# En Bloc Resection of Primary Large Esophageal Mucosa-Associated Lymphoid Tissue Lymphoma by Endoscopic Submucosal Dissection: A Case Report

**DOI:** 10.3389/fmed.2021.757485

**Published:** 2021-10-13

**Authors:** Yujia Xia, Yu Wang, Jian Han, Mei Liu

**Affiliations:** ^1^Department of Gastroenterology, Tongji Hospital, Tongji Medical College, Huazhong University of Science and Technology, Wuhan, China; ^2^Institute of Pathology, Tongji Hospital, Tongji Medical College, Huazhong University of Science and Technology, Wuhan, China

**Keywords:** esophagus, mucosa-associated lymphoid tissue lymphoma, endoscopic submucosal dissection, pathology, treatment

## Abstract

Treatment of mucosa-associated lymphoid tissue (MALT) lymphoma has recently received considerable attention. Here, we report a case of large esophageal MALT lymphoma that was successfully en bloc resected using endoscopic submucosal dissection (ESD). A 77-year-old woman was admitted to our hospital with progressive dysphagia for more than 2 months. Upper gastrointestinal endoscopy revealed a large rounded submucosal mass covered by normal mucosa, located at the lower esophagus. Endoscopic ultrasonography (EUS) showed a well-demarcated hypoechoic mass chiefly located in the esophageal wall, but the layers of the esophageal wall were not clear. ESD was performed for diagnostic and treatment purposes. No complications occurred during or after ESD. The resected specimen measured 4.3 cm × 2.8 cm × 1.5 cm. The histologic findings were diagnostic of esophageal MALT lymphoma. Infiltration of neoplastic cells in the lateral margins of the resected specimen was not observed. However, vertical margins showed an R1 situation and mild damage to the muscularis propria. After 3 months, her dysphagia disappeared. Additional radiation therapy was then administered. After 5 months, the patient was still under surveillance and free of recurrent disease. Resection with ESD of such a large mass of MALT in the esophageal region has rarely been reported before in the literature.

## Introduction

Esophageal lymphoma is usually secondary to the metastasis of lymph nodes from the cervical and mediastinal region or local invasion from the stomach (1), and thus primary esophageal lymphoma (PEL) is extremely rare, which accounts for <1% of cases of all primary gastrointestinal lymphoma (2). The clinical manifestations of PEL are non-specific, which may vary based on symptoms such as dyspepsia, dysphagia, nausea, vomiting, abdominal distention, weight loss, fever, and even epigastric pain to massive hemorrhage (3). The pathological subtypes of PEL are mainly represented by diffuse large B cell lymphoma (DLBCL) and mucosa-associated lymphoid tissue (MALT) lymphoma, and other B, T, or NK cell lymphoma and Hodgkin lymphoma in a few cases (4, 5). MALT lymphoma has the highest incidence in patients aged between 50 and 60 years (6), but it was observed that the incidence increased significantly in patients older than 40 years (7). To date, a few cases of esophageal MALT lymphoma have been reported in the literature. Due to the rarity, no standard treatment of primary esophageal MALT lymphoma has been established and its prognosis is unclear.

Here, we report a case of primary large MALT lymphoma of the esophagus that was successfully en bloc resected by endoscopic submucosal dissection (ESD) and diagnosed.

## Case Description

**Figure 1:**
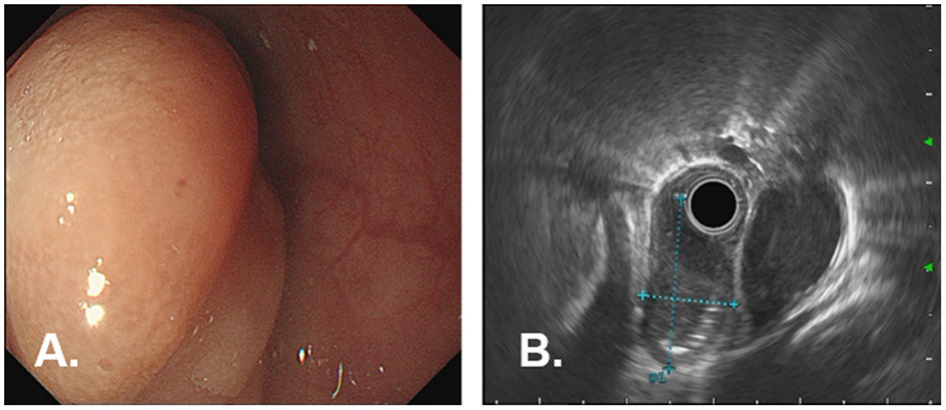
Endoscopic findings in a 77-year-old woman with dysphagia. **(A)** Upper gastrointestinal endoscopy showed a large, rounded mass with smooth normal overlying mucosa, which is seen extending longitudinally along the lower esophagus, 30–34 cm from the incisor teeth. **(B)** EUS shows a well-demarcated, hypoechoic mass in the esophagus wall, clearly margined from the surrounding adventitia.

**Figure 2:**
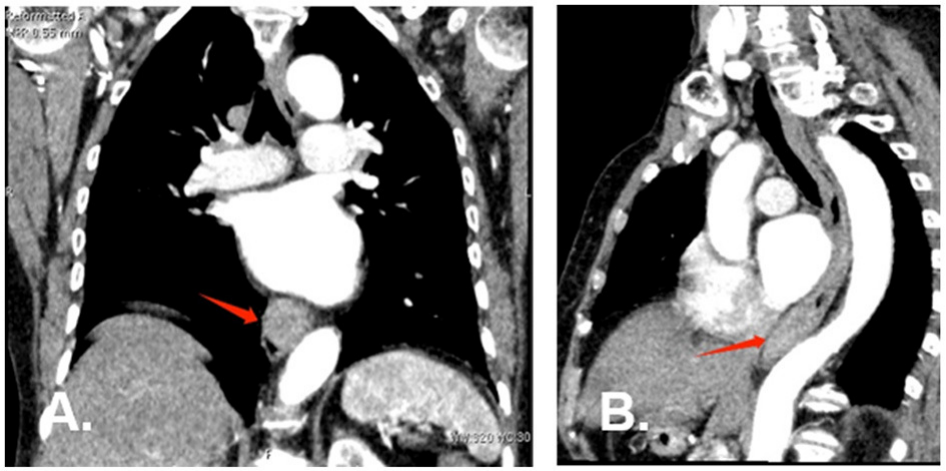
Chest contrast-enhanced computed tomography at diagnosis. **(A)** (Coronal plane) and **(B)** (Sagittal plane) CT scan revealed a well-defined homogeneous soft tissue mass at the lower esophagus (red arrow).

A 77-year-old woman was attended to our hospital for evaluation of an esophageal submucosal tumor (SMT) and complained of progressive dysphagia for more than 2 months. She had first noted intermittent difficulty in swallowing solids 2 months before the visit. Her symptoms worsened progressively over the past 1 month with difficulty in swallowing both solids and liquids. She had no significant weight loss during this time period. Her past medical history included a right buccal mass that had undergone resection 1 year ago. She had no history of any immunosuppressive disease, alcohol abuse, or smoking. The findings of physical examinations showed no abnormalities, and the superficial lymph nodes, liver, and spleen were not palpable. Laboratory tests revealed normal levels of blood routine, liver function, kidney function, and blood electrolytes. No hepatitis B surface antigen and hepatitis C virus antibodies were detected. No elevation of tumor markers or autoimmune antibodies was observed. Upper gastrointestinal endoscopy revealed a large rounded esophageal submucosal mass covered by normal mucosa, located at the lower esophagus, 30–34 cm from the central incisors (Figure 1A). Endoscopic ultrasonography (EUS) showed a well-demarcated hypoechoic mass chiefly located in the esophageal wall (Figure 1B), clearly separated from the surrounding adventitia. Findings on chest contrast-enhanced computed tomography (CT) revealed a well-defined homogeneous mass in the lower esophageal region, with size 18 mm × 28 mm (Figures 2A,B). Despite her age, in consultation with the patient, we chose and performed an ESD by injection-and-cut technique to completely remove the large esophageal lesion to allow for accurate histological diagnosis.

**Figure 3:**
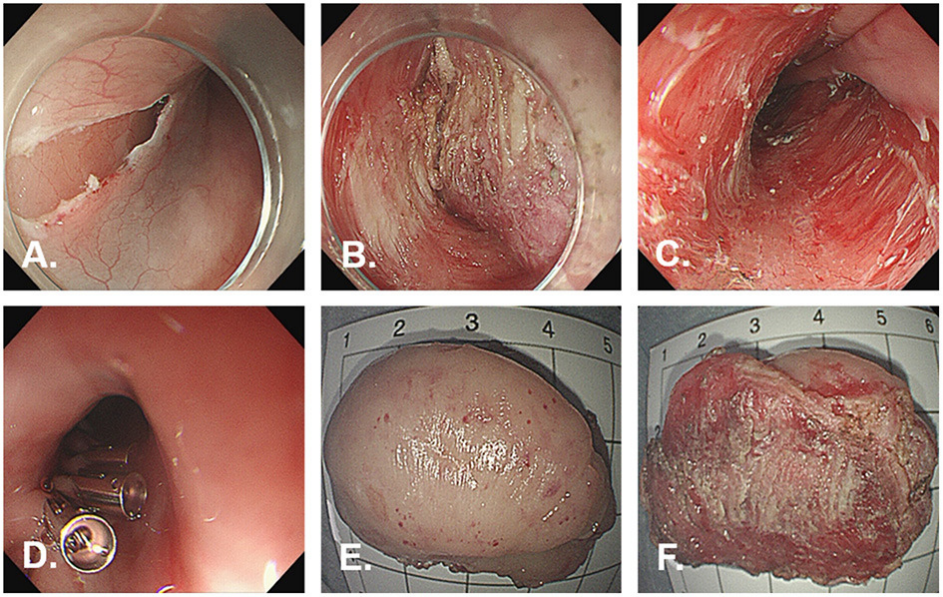
Images during endoscopic submucosal dissection. Endoscopic submucosal tunnel dissection is performed without any complication. **(A)** Initial incision of the mucosa after injection. **(B)** Exposure of the tumor. **(C)** The wound after ESD. **(D)** Complete closure of the mucosal incision site with endoclips. **(E)** On the external surface of the resected specimen. **(F)** On the cut surface of the resected specimen.

ESD was performed for diagnostic and treatment purposes. In this case, we used ESD-derived technique of submucosal tunneling endoscopic resection (STER). The submucosal injection was performed from the oral side at a distance of 3–5 cm from the tumor. Fluids were injected beneath the mucosa by a submucosal injection needle through the endoscopic channel to create a cushion. The fluid was a normal saline solution combined with 1:10,000 epinephrine and 1% methylene blue. A 2-cm longitudinal mucosal incision for a tunnel entry was made using a Dual knife. The submucosal layer was dissected using Dual knife and IT nano knife. Carbon dioxide was used for insufflation. After accomplishing the dissection, the lesion was removed using a basket and processed for histological evaluation. The dual knife was used for treating the possible vessels during the inspection. The mucosal incision site was closed with endoscopic clips (Figures 3A–D). The resected specimen measured 4.3 cm × 2.8 cm × 1.5 cm (Figures 3E,F).

**Figure 4:**
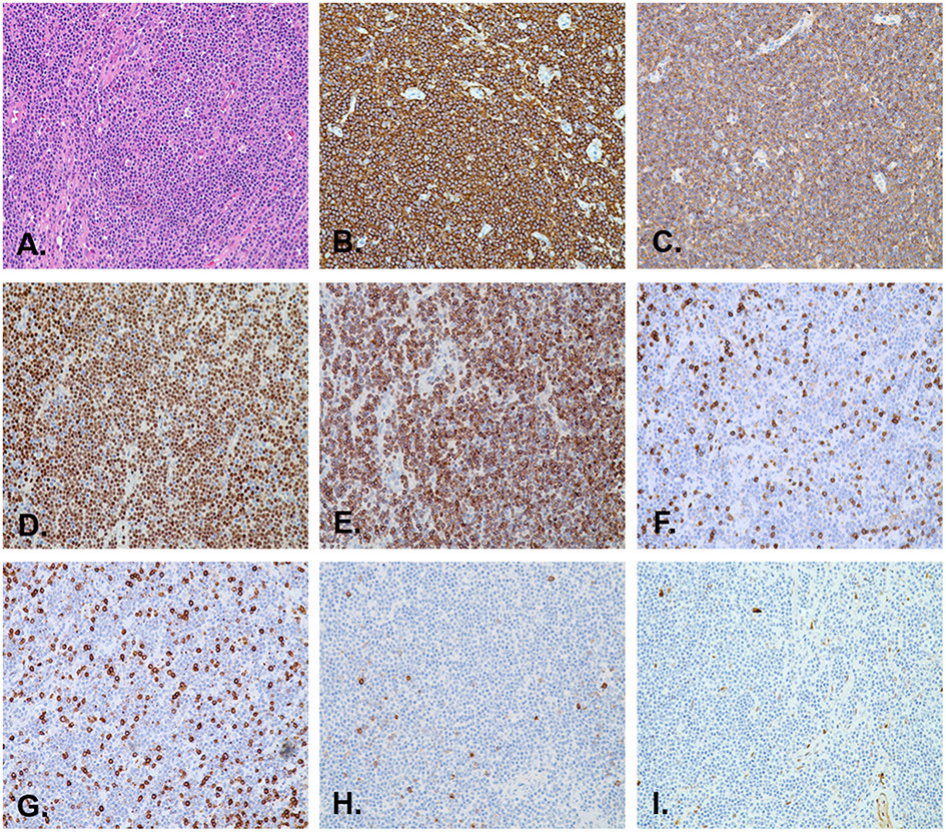
Pathological images of the resected specimen. **(A)** The histological section shows lymphoid hyperplasia in the lamina propria and submucosa (H&E stain, orig. mag. ×200). Immunohistochemistry revealed that the lymphoma cells are positive for CD20 **(B)**, CD19 **(C)**, PAX5 **(D)**, and BCL2 **(E)**, and negative for CD3 **(F)**, CD5 **(G)**, CD10 **(H)**, and cyclin D1 **(I)** (orig. mag. ×200).

ESD was completed without any complications. A broad-spectrum antibiotic and proton pump inhibitor were administered intravenously for the next 3 days after the procedure. The patient was fasting and receiving fluid therapy for 3 days. She was discharged 5 days after the surgery, and an oral proton pump inhibitor was prescribed for the next 4 weeks. The histopathological findings of the resected specimen showed infiltration of small- to medium-sized lymphoid cells with slightly irregular dark nuclei and abundant cytoplasm (Figure 4A). Neoplastic cells infiltrated the lamina propria to the submucosal layers. Infiltration of neoplastic cells in the lateral margins of the resected specimen was not observed. However, vertical margins showed an R1 situation and mild damage to the muscularis propria. Immunohistochemical studies revealed that the lymphoid cells were positive for CD20 (Figure 4B), CD19 (Figure 4C), PAX5 (Figure 4D), as well as BCL2 (Figure 4E), and negative for CD3 (Figure 4F), CD5 (Figure 4G), CD10 (Figure 4H), and cyclin D1 (Figure 4I). The percentage of tumor cells positive for Ki-67-staining was <5%, indicating few mitotic cells. The diagnosis of esophageal MALT lymphoma was confirmed based on these pathological features. The patient was not tested for Helicobacter pylori during hospitalization. Therefore, she was not treated for Helicobacter pylori eradication. During the follow-up visit, the patient complained that her 13 C-urea breath test result was negative for Helicobacter pylori in the past.

After 3 months, her dysphagia disappeared and a follow-up endoscopy showed no recurrence or complication at the ESD site, except for the presence of a scar. During the follow-up visit, the patient received additional radiation therapy according to the oncologist's suggestion. After 5 months, the patient was still under surveillance and free of recurrent disease.

## Discussion

Lymphoma arising in the esophagus is uncommon, accounting for <1% of patients with primary gastrointestinal lymphoma. Moriya et al. collected cases of PEL in stage I using the Lugano system staging and found that only 12 of the 37 cases (32.4%) were MALT lymphoma (8, 9). No finding from imaging was specific for the diagnosis of MALT lymphoma. Under barium swallow examination, PEL showed irregular filing defects due to segmental ulceration or narrowing and submucosal nodules which are similar to adenocarcinoma, esophageal varices, and achalasia (10, 11). CT findings show a thickened esophageal wall with a narrowed lumen that was not a target sign. PEL should be absent from cervical or mediastinal lymphadenopathy, so CT examination could exclude the involvement of lymph nodes in the cervical or mediastinal region (12). Endoscopic findings of PEL were variable and included submucosal nodular, polypoid growth, ulceration, and stenosis. EUS could detect structural changes of the digestive tract, which makes it valuable in assessing the depth of invasion, the extraluminal extent of the disease, or the extension in the lymph nodes. However, its findings were also non-specific, varying from anechoic, hypoechoic, or even hyperechoic masses (3, 13, 14).

MALT lymphoma appears in association with chronic inflammation induced by persistent infection and autoimmune diseases such as *Helicobacter pylori* (HP) infection and Hashimoto's thyroiditis, respectively (15, 16). Gastric MALT lymphomas without *t* (11; 18) translocation are well-known to be associated with HP infection, although there are very few cases of localized esophageal MALT lymphoma in HP-infected patients. Hence, HP infection was not a frequent marker for esophageal MALT lymphoma (17). Other factors such as the mechanical stimuli of food, hot water, chemicals in meals, other infections, and reflux esophagitis could also be involved in the development of primary esophageal MALT lymphoma. The underlying mechanisms of esophageal MALT lymphoma remained to be investigated.

The most frequent symptoms were dysphagia due to narrowing of the esophagus, epigastric pain, and weight loss. All of them were non-specific. Currently, because of the rarity of esophageal MALT lymphoma, a standard treatment for esophageal MALT lymphoma has not yet been established and recommended. Though radiotherapy is a treatment of choice for patients with large lesions in many other sites (18, 19), in esophageal MALT lymphoma, 64% of the cases were first treated with surgical resection or endoscopic resection (20, 21). In general, MALT lymphoma is not sensitive to chemotherapy, so chemotherapy is not recommended as the first-line treatment. In MALT lymphoma, the use of chemotherapy has been reserved for patients with disseminated disease or local treatment failure (22). ESD was confirmed as a useful therapeutic procedure for early gastric and esophageal cancers. The procedure also results in an improved differential diagnosis of malignant lymphoma occurring in digestive organs. To our knowledge, there have only been a few reports on ESD of early MALT lymphoma in the English literature (23–26). In the case reported here, ESD of the esophagus en bloc removed the large lesions and additional radiation was administered. If the MALT lymphoma is limited to the submucosa, ESD should be one of the most adequate and effective treatments. The indication of ESD for MALT lymphoma is limited to stage I, which means a tumor is located in the mucosa or submucosa without any lymphadenopathy. Compared to endoscopic mucosal resection (EMR), ESD has been shown to be superior because it is able to reach an en bloc resection and perform accurate histological examination (27). A pathological evaluation is extremely important in the diagnosis of lymphomas. The histological phenotype of a typical MALT lymphoma is positive for CD19, CD20, CD22, CD79a, and BCL2, and negative for CD3, CD5, CD10, CD23, and cyclin D1 (28–30).

In conclusion, although rare, primary esophageal MALT lymphoma should be considered in the differential diagnosis of esophageal SMT-like lesions. In addition, in cases in which the primary esophageal MALT lymphoma is confined to the deep mucosa and/or submucosa on EUS and the other sites are free of the disease, the lesion can be curatively removed by endoscopic resection. We report a case of successful ESD with primary esophageal MALT lymphoma. ESD may be a suitable and reasonable option as an attractive and less invasive local treatment for primary esophageal MALT lymphoma. The clinical profile of primary esophageal MALT lymphoma remains unclear, so it is important to accumulate more information on this rare entity.

## Data Availability Statement

The original contributions presented in the study are included in the article/supplementary material, further inquiries can be directed to the corresponding author/s.

## Ethics Statement

Ethical review and approval was not required for the study on human participants in accordance with the local legislation and institutional requirements. The patients/participants provided their written informed consent to participate in this study. Written informed consent was obtained from the individual(s) for the publication of any potentially identifiable images or data included in this article.

## Author Contributions

YX and ML were involved in the study design and concept. YW was involved in data acquisition. JH revised the manuscript for critical intellectual content. All authors have read and approved the final manuscript and were involved in the review and editing of the manuscript.

## Funding

This work was supported by the National Natural Science Foundation of China (NSFC 81700515).

## Conflict of Interest

The authors declare that the research was conducted in the absence of any commercial or financial relationships that could be construed as a potential conflict of interest.

## Publisher's Note

All claims expressed in this article are solely those of the authors and do not necessarily represent those of their affiliated organizations, or those of the publisher, the editors and the reviewers. Any product that may be evaluated in this article, or claim that may be made by its manufacturer, is not guaranteed or endorsed by the publisher.
